# Identification of autophagy-related key biomarkers in caerulein induced acute pancreatitis: In silico and in vivo study

**DOI:** 10.1371/journal.pone.0344110

**Published:** 2026-03-27

**Authors:** Xinzhuang Shen, Jie Yao, Zongke Wang, Renhua Chen, Laian Li, Xiangjun Xu, Yongming Huang

**Affiliations:** 1 Department of General Surgery, Affiliated Hospital of Jining Medical University, Jining, People’s Republic of China; 2 Jining Hospital of Traditional Chinese Medicine, Xiyuan Hospital of China Academy of Chinese Medical Sciences, Jining, People’s Republic of China; 3 Department of Critical Care Medicine, Affiliated Hospital of Jining Medical University, Jining, People’s Republic of China; 4 Key Laboratory of Precision Oncology in Universities of Shandong, Institute of Precision Medicine, Jining Medical University, Jining, China; 5 Key Laboratory of Hepatosplenic Surgery, The First Affiliated Hospital of Harbin Medical University, Harbin, People’s Republic of China; Central South University, CHINA

## Abstract

**Background:**

Dysregulated autophagy is known to play a crucial role in the pathogenesis of Acute pancreatitis (AP). However, its specific mechanisms remain unclear. The present study aimed to elucidate the mechanism of autophagy-related genes (ARGs) by bioinformatics analysis and experimental validation in AP.

**Methods:**

GSE109227, GSE65146, and GSE3644 were obtained from GEO database and underwent standardization and principal component analysis. ARGs were sourced from the Human Autophagy Database. Differentially expressed genes (DEGs) were intersected with ARGs to identify differentially expressed autophagy-related genes (DEARGs) associated with AP. Subsequently, functional enrichment analysis, Gene Set Enrichment Analysis, and Gene-gene interaction networks were performed. The expression of DEARGs at the mRNA level was verified using the GSE121038 dataset, and protein expression levels were examined in animal model by western blot. Potential regulatory chemicals or compounds affecting the expression of DEARGs were predicted using the Comparative Toxicogenomics Database (CTD).

**Results:**

We screened seven key biomarkers (Sesn2, Kras, Hmox1, Cast, Nfe2l2, Npc1, and Cdkn1a) from the GSE109227, GSE65146, and GSE3644 datasets. These biomarkers showed higher levels of mRNA expression compared to the control group. Additional testing using the GSE121038 dataset showed that only Npc1 mRNA level was not significantly different (P > 0.05). The protein expression levels of Sesn2, Hmox1, and Kras aligned with mRNA expression, while Nfe2l2, Cast, and Npc1 showed opposing patterns. No differences were detected in Cdkn1a. GSEA revealed significant enrichment of the ARG set in AP samples across the three GEO datasets. Functional enrichment analysis indicated the DEARGs primarily participate in the regulation of autophagy and macroautophagy. Several chemicals or compounds were projected to modulate DEARGs expression, notably acetaminophen.

**Conclusion:**

Our research further highlights the significant interaction between autophagy and AP. Notably, Sesn2, Kras, Hmox1, and Nfe2l2 exhibited significant expression differences during acute pancreatitis, suggesting they may serve as potential biomarkers for the condition.

## Introduction

Acute pancreatitis (AP) is a complex inflammatory condition characterized by pancreatic autodigestion, leading to severe local and systemic inflammation [1]. The pathology of AP involves a cascade of inflammatory responses initiated by necrotic cells releasing inflammatory factors, resulting in systemic inflammatory response syndrome (SIRS) and potentially progressing to multiple organ dysfunction syndrome (MODS) [2], with no established effective treatments to date. Although the precise mechanisms of AP pathogenesis remain unclear, the formation of cytoplasmic vacuoles and the premature activation of trypsinogen within acinar cells are recognized as early features [3,4]. During the development of acute pancreatitis, abnormalities in the activity of various signaling pathways, including autophagy, regulate these two features [5]. Single-cell data studies confirm that impaired autophagy plays a central role in its pathogenesis [6]. Current investigations aim to decipher the specific role of autophagy in AP.

Autophagy is a cellular process whereby damaged or aging proteins and organelles are transported to lysosomes for degradation [7]. This process encompasses several subtypes, including microautophagy, macroautophagy, and chaperone mediated autophagy, all essential for intracellular homeostasis. Macroautophagy, hereafter referred to as autophagy, is extensively studied and uniquely observed both in the normal and inflamed exocrine pancreas [8]. Emerging evidence links the maladaptation of autophagy to the two critical early features of AP. For instance, deregulated activation of autophagy can jeopardize pancreatic acinar cells, aggravating pancreatic damage and intensifying pancreatitis symptoms [9]. Animal models of acute pancreatitis have demonstrated that deletion of ATG5 lessens autophagy severity and diminishes trypsinogen activation [10]. Furthermore, during AP onset, pancreatic α7 nicotinic acetylcholine receptor (^9^α7nAChR) signaling is believed to enhance TFEB expression, thereby increasing acinar cell autophagy [11]. Liang Ji’s research implicated excessive autophagy activity, linked with ATG7 expression levels, in exacerbating AP through upregulation of CAMKII-regulated necrosis, mediated by a decrease in miR-30b-5p levels [12]. Mareninova OA’s findings suggest that acute pancreatitis features impaired cathepsin processing and activity, slowing autophagic flux, evidenced by vacuole accumulation and trypsinogen activation within acinar cells [13]. Yet, numerous autophagy-related genes with roles in AP pathogenesis have not been discovered, warranting further research.

To our knowledge, no previous studies have integrated bioinformatics and experimental validation to investigate the role of autophagy-related genes in AP. Therefore, we undertook a differential expression analysis across three GEO datasets to identify differentially expressed genes. GSEA analysis of the autophagy-related dataset indicated an upregulation of the autophagy pathway in AP. We identified seven key biomarks and conducted a functional enrichment analysis. These findings were validated using the GSE121038 dataset and an animal model induced by caerulein and lipopolysaccharide. Western Blot confirmed the protein expression levels of the key biomarks in AP tissue. These insights elucidate multiple genes’ involvement in modulating autophagy during AP and contribute valuable knowledge for clinical diagnosis and therapeutic interventions.

## Materials and methods

### Autophagy-related genes datasets and microarray data

Since all data in this study were obtained from mice, we selected mouse as the species evidence in The Human Autophagy Modulator Database (HAMdb, http://hamdb.scbdd.com) [14] and obtained a total of 212 autophagy-related genes (ARGs). The mRNA expression profile datasets of GSE109227, GSE65146, GSE3644 and GSE121038 were downloaded from GEO (http://www.ncbi.nlm.nih.gov/geo/). All samples were obtained from pancreatic tissues of wildtype mice. Animal model of AP were induced by injecting intraperitoneally with caerulein and Lipopolysaccharide, and control group were induced by injecting with normal saline. Table 1 describes the dataset information.

**Table 1:** The GEO dataset information.

DataSets	Platform	AP samples	Control samples	Gene Expression Omnibus Sample	Gene Expression Omnibus Sample
GSE3644	GPL339	n = 3	n = 3	GSM84552 GSM84553 GSM84554	GSM84549 GSM84550 GSM84551
GSE65146	GPL6246	n = 3	n = 5	GSM1588094 GSM1588095 GSM1588096	GSM1588086 GSM1588087 GSM1588088 GSM1588089 GSM1588090
GSE109227	GPL6246	n = 6	n = 5	GSM2935594 GSM2935595 GSM2935596 GSM2935597 GSM2935598 GSM2935599	GSM2935589 GSM2935590 GSM2935591 GSM2935592 GSM2935593
GSE121038	GPL10787	n = 4	n = 3	GSM3424908 GSM3424909 GSM3424910 GSM3424911	GSM3424905 GSM3424906 GSM3424907

### Screening of DEGs by GEO2R

The GEO2R (http://www.ncbi.nlm.nih.gov/geo/geo2r), is an online data analysis tool, which was used to identify the DEGs between AP and control groups in mice [15]. The expression matrix of GSE109227, GSE65146 and GSE3644 datasets were normalized in GEO2R, and then principal component analysis (PCA) was performed between AP group and the Control group [16]. Genes with an adjusted P-value <0.05 and |logFC| > 1.0 were considered as differentially expressed genes（DEGs）. The ggplot2 package was used to visualize the difference analysis results.

### Identification and differentially expressed analysis of DEARGs

By Venn intersection, we screened for differentially expressed autophagy-related genes (DEARGs). The volcano plot analysis were performed by Hiplot (https://hiplot.com.cn), a comprehensive web platform for scientific data visualization [17]. Box plot were conducted using “ggplot2” packages of R software.

### Fuctional enrichment analysis and gene set enrichment analysis (GSEA)

Gene ontology (GO, http://www.geneontology.org/) term and Kyoto Encyclopedia of Genes and Genomes (KEGG, http://www.genome.jp/kegg) pathway enrichment analysis were conducted in R software using the package “clusterProfiler” [18]. The GO analysis consisted of cellular component (CC), biological process (BP) and molecular function (MF). We used threshold p-value 0.05. GSEA was plotted by https://www.bioinformatics.com.cn, an online platform for data analysis and visualization.

### Gene–gene interaction analysis of DEARGs

GENEMANIA is a tool database that constructs interactive networks by retrieving and identifying genes similar to the target gene (http://genemania.org/search/) [19]. In this study, we used GENEMANIA to construct the gene–gene interaction network (GGI) and evaluated the functions of DEARGs.

### Drug-gene interaction network analysis

Chemotherapeutic drug-gene interaction networks were constructed by using the Comparative Database, an online database providing information on gene product interactions with chemotherapeutic drugs, and their relationship to disease [20]. The networks were visualized by Cytoscape software 3.7.2.

### Molecular docking

The 3D structure of acetaminophen was downloaded from the PubChem database (CAS: 103-90-2) and saved in sdf format. The three-dimensional structures of SESN2 (PDB ID: 5DJ4), KRAS (PDB ID: 1D8D), NPC1 (PDB ID: 3GKI), CDKN1A (PDB ID: 2ZVV), HMOX1 (PDB ID: 1NI6), and NFE2L2 (PDB ID: 1ITV) were obtained from the Protein Data Bank (https://www.pdbus.org/). The three-dimensional structures of CAST (ID: P20810) was obtained from the AlphaFold Protein Structure Database (https://alphafold.ebi.ac.uk/). Water molecules and organic matter were removed from the target protein using Notepad2, and the modified protein was subsequently imported into AutoDockTools 1.5.6 for further processing. Molecular docking was performed using AutoDock Vina, and the docking conformation that exhibited the minimal binding energy was designated as the optimal binding pose. The docking results were visualized using PyMOL 2.6 [21].

### Animals and handling methods

Eighteen male C57 mice weighing 18–21 g each were obtained from Jinan Pengyue Laboratory Animal Breeding Co. Experimental AP was induced by caerulein as described in previous studies [22]. All procedures were meticulously performed in accordance with the ethical standards of the Animal Ethics Committee of Jining Medical College (Approval No: 2023B069), and the experimental reporting followed the ARRIVE guidelines. These mice were then systematically grouped into three categories. The first group, known as the saline control group (NS group), consisted of six mice treated with saline. The second group, termed the caerulein group (CAE group), had six mice receiving 50ug/kg of caerulein every hour, ten times in total. The third group, the caerulein + lipopolysaccharide group (CAE + LPS group), included six mice given 50ug/kg caerulein hourly for six instances, followed by a 10 mg/kg dose of lipopolysaccharide an hour posts the final caerulein administration. All mice were anaesthetized via intraperitoneal injection of Avertin (Sigma-Aldrich, Shanghai, China) at 4 mg/10 g mice weight. Immediately following this, blood samples were collected via cardiac puncture. Prior to experiment, it is imperative that the cardiac puncture technique and operational procedures are thoroughly mastered in order to enhance success rates. In order to minimize suffering, all animals were euthanized by cervical dislocation prior to the collection of pancreatic tissue. The organ’s head was preserved in 4% paraformaldehyde, its mid-section was weighed in its wet state, and the tail portion underwent lysis for protein extraction, which was then analyzed using western blot.

### Measurement of Serum amylase activities

After drawing blood from one side of the mouse’s eye, the sample was refrigerated at 4°C for 30 minutes. Following the refrigeration of the blood sample, it was centrifuged under the same conditions (4°C and 4000 rpm) for 30 minutes. After centrifugation, the serum layer on top was collected and stored at −80°C for future use. The amylase content in the serum was then measured using the amylase activity assay kit (MAK009) from Sigma.

### To detect the wet/dry weight ratio of pancreatic tissue

To assess pancreatic edema, the water content was measured. Wet weight refers to the weight of the freshly harvested pancreas. Dry weight refers to the weight after 72 h of desiccation at 60 °C.

### Histomorphological examination of the pancreas

Pancreatic head tissue was first fixed in 4% formalin for 24 hours. After fixation, they were dehydrated and embedded in paraffin. The embedded tissues were then sectioned into 4μm thick slices. These slices were stained with H&E, subsequently examined, and scanned.

### Western blotting

The tail portion of the removed mouse pancreas was placed in a mortar, frozen with liquid nitrogen and then rapidly ground. The ground tissue samples were washed twice with 1 × PBS and added to RIPA protein lysate overnight in the refrigerator at 4°C. The protein concentration was measured and quantified by the BCA method, and after loading approximately 7ug of protein on a polyacrylamide gel, the electrophoresis was performed at room temperature using 100 V. Then, the proteins were transferred to PVDF membranes with transfer buffer for 70 minutes at 4°C and then closed with 5% skimmed milk for 1 hour at room temperature. The rabbit anti-Calpastatin (12250–1-AP, Proteintech, WuHan, China), rabbit anti-Ho1 (10701–1-AP, Proteintech, WuHan, China), rabbit anti-Sestrin2 (10795–1-AP, Proteintech, WuHan, China), rabbit anti-Npc1 (13926–1-AP, Proteintech, WuHan, China), rabbit anti-Nfe2l2 (16396–1-AP, Proteintech, WuHan, China), rabbit anti-P21, mouse anti-Kras (sc-30, Santa Cruz, CA, USA) and mouse anti-β-actin (sc-47778, Santa Cruz, CA, USA) were used separately in Tris buffered physiological saline (TBST) overnight at 4°C and incubated with the corresponding secondary antibodies for 1h the next day. Finally, they were washed 3 times in TBST and analyzed by enhanced chemiluminescence.

### Cell activity measurement

AR42J cells were seeded into a 96-well plate at a density of 1 × 10⁴ cells per well. The cells were then treated with acetaminophen acetaminophen at concentrations ranging from 0 to 1800 µM for 24 hours. The Cell Counting Kit-8 (Cat. # CK04, Dojindo) was utilized to quantify cell activity in accordance with the manufacturer’s instructions. The absorbance was subsequently measured at 450 nm using an automated multi-scanner and the half-maximal inhibitory concentration (IC50) value was calculated.

### Statistical analysis

Statistical analyses of gene expression levels were performed using the R (version 4.0.0). In instances where the samples satisfied the normality test criteria, an Independent-Samples T-test was employed to analyse the two groups. Conversely, the Mann–Whitney U test was applied to analyse the two groups when the samples did not satisfy the aforementioned criteria. Furthermore, in instances where the samples satisfied the normality test criteria, one-way ANOVA was applied to analyse the multiple groups. In the event that the samples did not satisfy the normality test, the Kruskal–Wallis test was employed for multiple groups.

## Results

### Data processing and principal component analysis (PCA)

**Fig 1:**
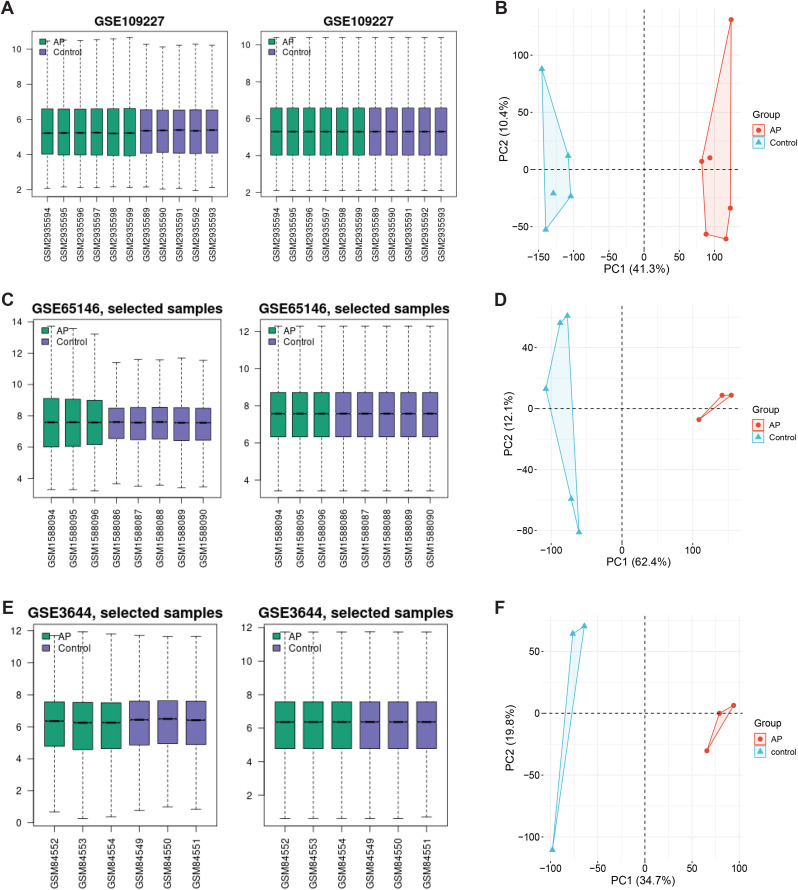
Normalized gene expression and principal component analysis. **(A)** The normalization of GSE109227 dataset. **(B)** The principal component analysis of GSE109227. **(C)** The normalization of GSE65146 dataset. **(D)** The principal component analysis of GSE65146. **(E)** The normalization of GSE3644 dataset. **(F)** The Principal component analysis of GSE3644. AP, acute pancreatitis.

We initially conducted normalization of the data across three datasets (GSE3644, GSE65146, and GSE109227) using the GEO2R, as depicted in Fig 1A, C, and E. Subsequently, a principal component analysis confirmed the clustering and demonstrated the reliability of the datasets (Fig 1B, D, and F).

### Screening and GO enrichment analysis of differentially expressed genes (DEGs)

**Fig 2:**
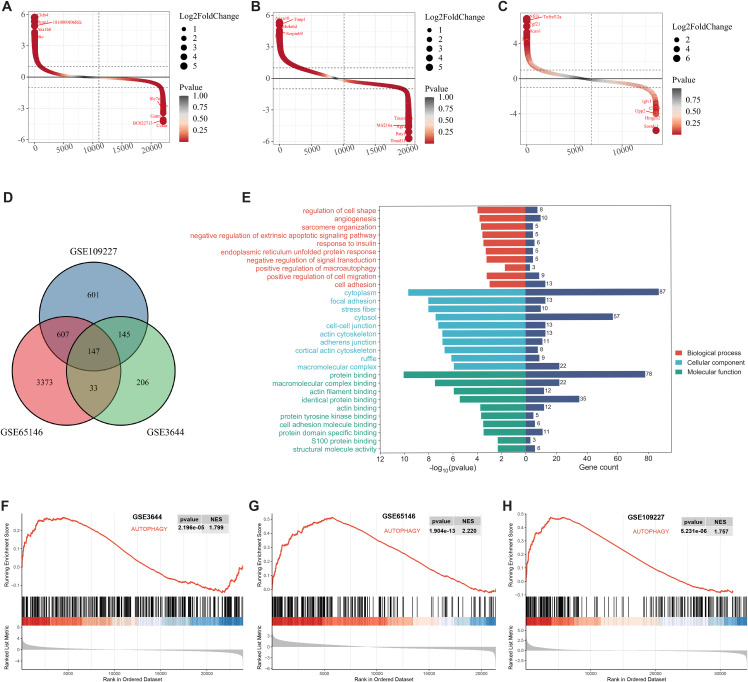
Identification of differentially expressed genes (DEGs) and GO analysis. Ranking plots for DEGs from three GEO datasets. Genes were sorted based on log2(FC) values, and DEGs with log2(FC) > 1 were selected. **(A)** Ranking of differentially expressed genes in GSE109227. **(B)** Ranking of differentially expressed genes in GSE65146. **(C)** Ranking of differentially expressed genes in GSE3644. The top 10 genes with the highest differential expression are marked in the plots. **(D)** Common DEGs identified by intersecting the three GEO datasets. **(E)** GO analysis of DEGs. GSEA analysis of ARG sets in the GEO dataset. **(F)** GSEA on ARG set in GSE3644. **(G)** GSEA on ARG set in GSE65146. **(H)** GSEA on ARG set in GSE109227. GSEA, gene set enrichment analysis; ARG, autophagy-related genes; Pval, P-value; NES, normalized enrichment score.

Applying a significance threshold of P < 0.05 and |logFC| > 1.0, we employed the LIMMA package to identify differentially expressed genes (DEGs), with visualization facilitated by the ggplot2 package (Fig 2A-C). We conducted an intersection analysis to screening DEGs common to three datasets, finding 147 shared DEGs (Fig 2D). The functional potential of 147 DEGs was investigated through GO enrichment analyses. Results demonstrated that these DEGs had significant enhancements in the regulation of cell shape, negative regulation of the extrinsic apoptotic signaling pathway, and positive regulation of macroautophagy. Relative to cellular components, enhancements were noted in the cytoplasm, focal adhesions, stress fibers, cytosol, and cell-cell junctions. Concerning molecular function, a significant enrichment was observed in protein binding, including macromolecular complex, actin filament, identical protein, actin, and protein tyrosine kinase (Fig 2E). Go enrichment analysis results indicate that macroautophagy is associated with acute pancreatitis.

### Relationship between autophagy and AP

To explore the role of the autophagy pathway in the pathogenesis of acute pancreatitis, we performed gene set enrichment analysis (GSEA). The autophagy-related gene set demonstrated significant enrichment in acute pancreatitis samples across three GEO datasets: GSE109227, GSE65146, and GSE3644 (Fig 2F-H). These findings indicate a substantial association between autophagy and the development of acute pancreatitis.

### Identification and biological function enrichment analysis of DEARGs

**Fig 3:**
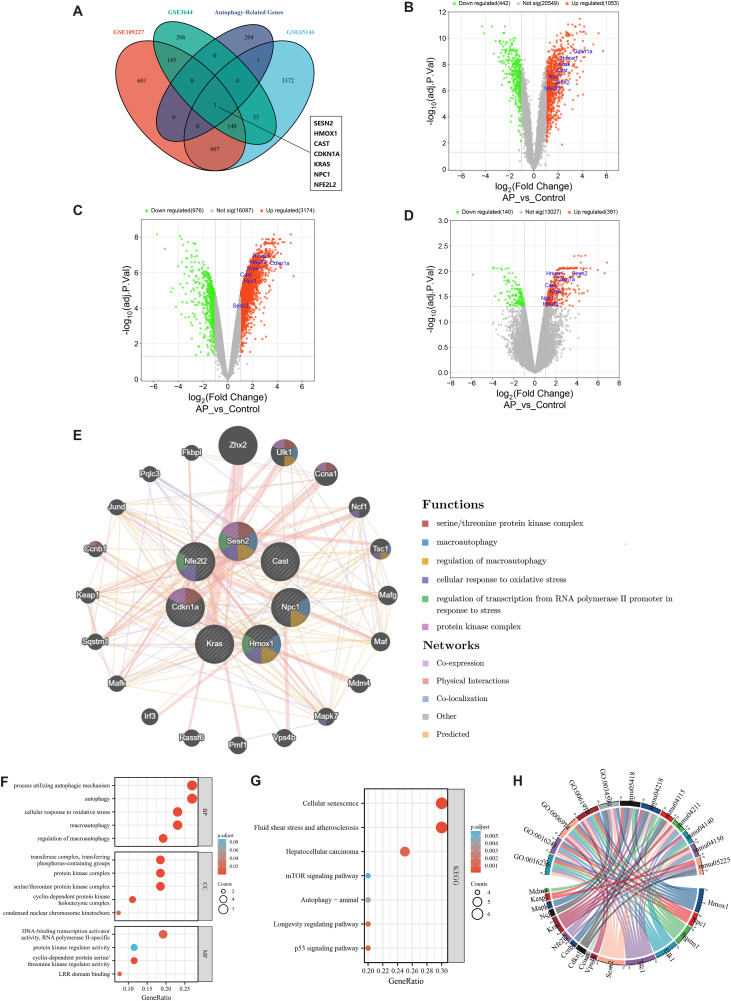
Identification of differentially expressed autophagy-related genes (DEARGs) and functional analysis. **(A)** DEARGs determined by intersecting DEGs with autophagy-related genes. **(B)** The volcano of DEARGs in GSE109227. **(C)** The volcano of DEARGs in GSE65146. **(D)** The volcano of DEARGs in GSE3644. **(E)** Gene-gene interaction network of DEARGs. Each node represents a gene, and the size of the node indicates the strength of the interaction. The color of the lines connecting nodes represents the type of gene interaction. The color of the nodes indicates the possible function of each gene. **(F)** GO enrichment analysis of DEARGs and 20 co-expressed genes. **(G)** KEGG enrichment analysis of DEARGs and 20 co-expressed genes. **(H)** Bubble diagram of GO enrichment term.

Based on species evidence for mouse, we obtained 212 autophagy-related genes from the Human Autophagy Database (S1 Table). Further intersection revealed seven DEARGs (Sesn2, Kras, Hmox1, Cast, Nfe2l2F, Npc1, Cdkn1a) associated with acute pancreatitis, as shown in the Venn diagram (Fig 3A). The volcano plot showed that the DEARGs were upregulated in all three GEO datasets (Fig 3B-D). Functional analysis was performed on the identified DEARGs. These genes were analyzed using the GeneMANIA database to construct protein interaction networks; consequently, 20 interacting genes were identified. Functional analysis indicated a significant enrichment of these genes in both macroautophagy and regulation of macroautophagy, as depicted in Fig 3E. Moreover, GO enrichment analysis of biological processes further revealed a preponderance of genes involved in autophagy, macroautophagy, and regulation of macroautophagy (Fig 3F). Additionally, KEGG enrichment analysis established a strong association of these genes with cellular senescence, fluid shear stress and atherosclerosis, and autophagy in animals (Fig 3G, H). These findings indicate that the identified DEARGs may play a crucial role in the pathogenesis of AP through the modulation of autophagy.

### Validation of DEARGs

**Fig 4:**
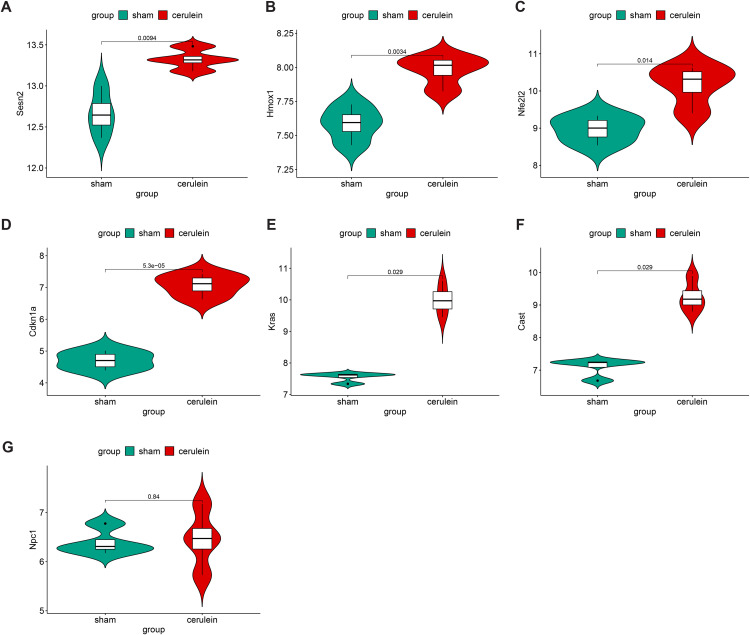
Validation of the mRNA expression of 7 DEARGs in GSE121038. The mRNAs of 6 DEARGs were significantly increased. (A) Sesn2, (B) Hmox1, (C) Nfe2l2F, (D) Cdkn1a, (E) Kras, (F) Cast. (G) Npc1 was not statistically significant compared to the sham group.

**Fig 5:**
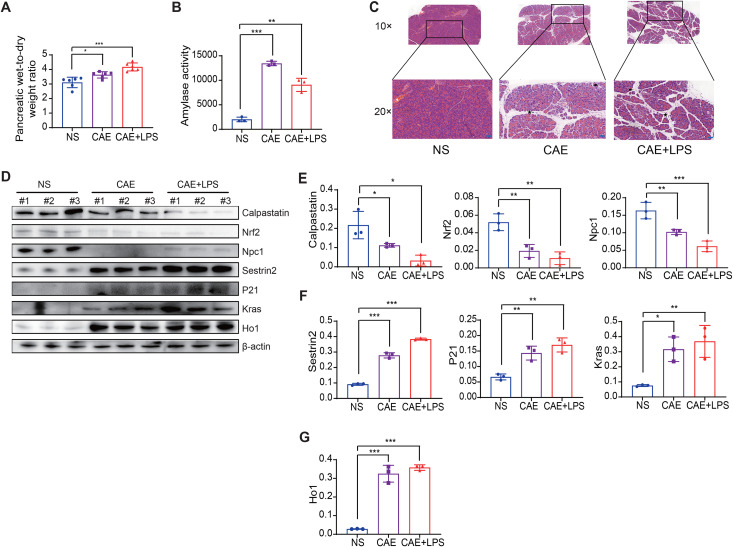
Animal model of acute pancreatitis was successfully constructed. **(A)** Pancreatic wet-to-dry weight ratio. **(B)** Serum amylase levels. **(C)** Representative images of pancreatic damage by H&E staining. Arrows indicate inflammatory cells, and asterisks indicate edematous stroma. **(D)** Western blot analysis was performed to assess the expression levels of DEARGs. (E) calpastatin, Nrf2, Npc1, **(F)** Sestrin2, p21, Kras, and **(G)** Ho1. Data shown are means ± SEM. NS: saline control, CAE: Caerulein, LPS: Lipopolysaccharides. *P < 0.05; **P < 0.01; ***P < 0.001; ns, no significant difference.

To validate the mRNA expression levels of seven candidate biomarkers, we analyzed data from an independent mouse model dataset (GSE121038) in the GEO. Fig 4A-F demonstrated that, in comparison with the sham group, mRNA expression levels of six biomarkers were significantly elevated in the AP group (P < 0.05). However, no statistically significant difference was observed in NPC1 mRNA expression, with P > 0.05 (Fig 4G). Subsequently, we induced AP and SAP in mouse models using caerulein and lipopolysaccharide, and quantified the protein expression levels in the pancreatic tissues. The outcomes indicated a marked increase in pancreatic edema and serum amylase in the model group compared to NS group (Fig 5A, B). Histological evaluation of pancreatic specimens revealed diffuse lobular expansion and disruption of pancreatic architecture, accompanied by substantial inflammatory cell infiltration and hemorrhagic necrosis (Fig 5C), thus confirming the successful establishment of the model. The results of the Western blot analysis indicated that, in comparison with the control group, the protein expression levels of Sesn2, Cdkn1a, Kras, and Hmox1 were significantly increased in the acute pancreatitis animal model (Fig 5D, F, and G). Conversely, Cast, Nfe2l2, and Npc1 exhibited a marked reduction in protein expression levels (Fig 5D, E). These results substantiate the association between autophagy-related genes and the development of AP.

### Drug-gene interaction network analysis

**Fig 6:**
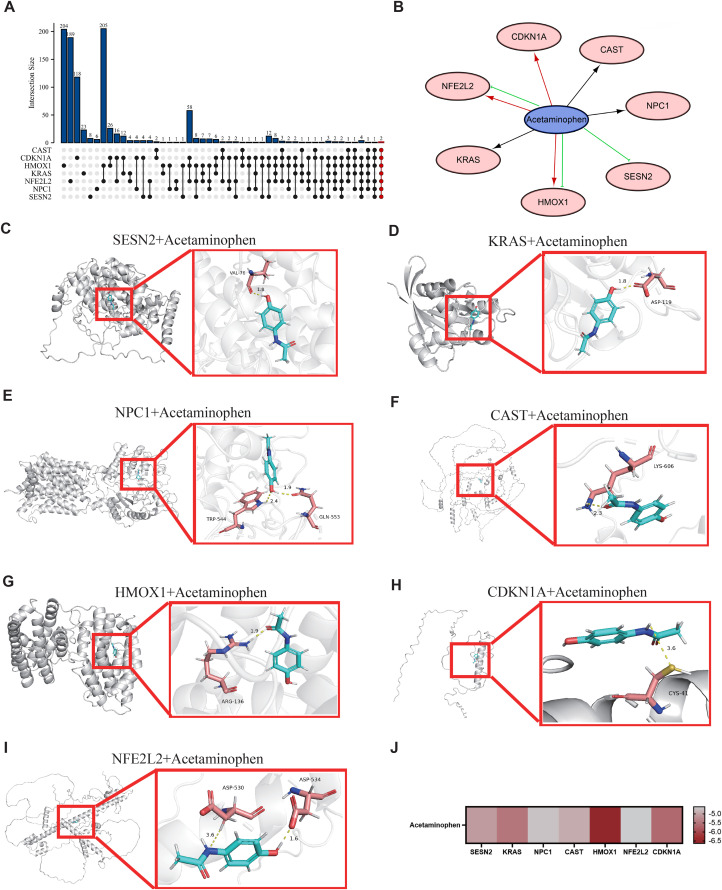
Using the CTD database to construct a drug-gene interaction network with 7 DEGARs. **(A)** An UpSetR plot shows that there are 2 drugs that regulate all genes. **(B)** Represents the relationship between existing drugs and the expression levels of DEARGs. Red and green arrows indicate that drugs will increase or decrease the expression of genes, respectively. Black arrows indicate that drugs will affect the expression of genes. Binding of potential drugs to DEARGs by molecular docking. **(C)** Binding mode of Acetaminophen to SESN2. **(D)** Binding mode of Acetaminophen to KRAS. **(E)** Binding mode of Acetaminophen to NPC1. **(F)** Binding mode of Acetaminophen to CAST. **(G)** Binding mode of Acetaminophen to HMOX1. **(H)**Binding mode of Acetaminophen to CDKN1A. **(I)** Binding mode of Acetaminophen toNFE2L2. **(J)** Heatmap of Docking Score. Compound is shown as cyan sticks. The key residues are shown as brick red sticks. Hydrogen bonds are shown as yellow dashed lines.

To elucidate the intricate information within the interactions between DEARGs and various compounds or drugs, we established a network detailing compound-protein interplay utilizing data from the Comparative Toxicogenomics Database (CTD). Acetaminophen (APAP) and vehicle emissions were identified as having interactive effects on the mRNA expression of all seven DEARGs (Fig 6A, S2 Table and S3 Table). The interaction results indicate that APAP either increases or decreases the expression levels of Hmox1 and Nfe2l2 mRNA. APAP reduced the level of Sesn2 mRNA expression. It also increased the expression of Cdkn1a. However, the exact regulatory effects of APAP on the mRNA expression of Kras, Cast, and Npc1 remain ambiguous (Fig 6B). We verified the binding energy and interaction patterns between APAP and DEARGs by molecular docking techniques. The results showed that the drugs bound to their protein targets through visible hydrogen bonds and strong electrostatic interactions (Fig 6C-I). The thermograms show that HMOX1 has the lowest binding energy of −6.632 kcal/mol, indicating highly stable binding (Fig 6J). Computational predictions suggest that APAP may play a role in drug-induced pancreatitis by affecting the autophagy pathway.

### The Effect of Acetaminophen on DEARGs in AR42J cells treated with caerulein

**Fig 7:**
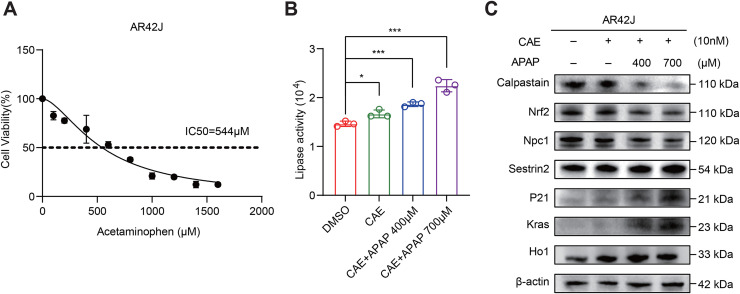
Effects of Acetaminophen on DEARGs in AR42J Cells Treated with caerulein. **(A)** AR42J cells were exposed to acetaminophen at concentrations ranging from 0 to 1800 µM for 24 hours. Cell viability was assessed using the CCK-8 assay, and the half-maximal inhibitory concentration (IC50) values were calculated. **(B)** Lipase activity was measured in different cell groups using a lipase assay kit. N = 3, *p < 0.05, **p < 0.01. **(C)** Western blot analysis was performed to assess the expression levels of calpastatin, Nrf2, Npc1, Sestrin2, p21, Kras, and Ho1, with β-actin as the loading control.

To further investigate the relevance of APAP in acute pancreatitis, an acute pancreatitis model was established using AR42J cells. Firstly, cell viability decreased in a dose-dependent manner, with an CCK8 assay-determined IC50 value of 544 μM (Fig 7A). Treatment of AR42J cells with caerulein resulted in elevated amylase levels. Concurrently, APAP induced a dose-dependent increase in amylase (Fig 7B), suggesting that excessive APAP may contribute to the development of acute pancreatitis. Furthermore, the results of the Western blot analysis showed that APAP affects the protein expression levels of seven biomarks in acute pancreatitis (Fig 7C). These genes are Sesn2, Kras, Hmox1, Cast, Nfe2l2, Npc1, and Cdkn1a.

## Discussion

Acute pancreatitis (AP) is an injurious inflammatory disease caused by the dysfunction of the exocrine pancreas. As a prevalent condition in general surgery, AP lacks effective treatments to curb the deterioration of pancreatic function [23]. Notably, the dysregulation of lysosomal pathways, autophagy, and inflammatory responses plays a crucial role in the pathogenesis of AP [24]. However, the complexity of autophagic processes presents challenges for research, as both hyperactivation and functional impairment of autophagy are implicated in the disease [25]. Consequently, additional research and validation are imperative to elucidate autophagy’s potential involvement in the pathomechanism of AP.

Recent advancements in bioinformatics analyses have become pivotal in the identification of potential biomarkers for various diseases [26]. Extensive studies have delved into the pathogenesis of AP, uncovering genes with therapeutic potential through expression profiling [27–29]. To our knowledge, only one study has used bioinformatics to analyze the role of autophagy-related ceRNA networks in AP at the molecular mechanism level. This notable study constructed an lncRNA-miRNA-mRNA regulatory framework by isolating lncRNAs intricately linked to m6A modifications in AP, shedding light on their regulatory capacity over autophagic processes and the disease’s progression [30]. Nonetheless, the molecular underpinnings of autophagy-related genes in AP have not been extensively examined through bioinformatics. In our research, we analyzed four GEO datasets to discern DEGs, thereby bolstering the study’s validity. GSEA corroborated a profound connection between autophagy and AP. Employing the HAMdb database, we identified seven DEARGs (Sesn2, Kras, Hmox1, Cast, Nfe2l2, Npc1, Cdkn1a) as pivotal in AP. While protein expressions of Sesn2, Hmox1, and Kras were consistent with their mRNA levels as validated by animal models. The absence of disparity in Cdkn1a protein levels between experimental cohorts could be attributed to individual variances and a limited sample size, warranting further investigation. Functional enrichment analyses indicated that these key genes predominantly participate in autophagy and macroautophagy regulation. Thus, elucidating the multifaceted roles of these genes will significantly contribute to our understanding of their involvement in autophagy and enhance the study of AP.

Sestrin2 (Sesn2) is a stress-responsive protein that experiences a marked increase in expression in response to a multitude of stressors, predominantly orchestrating the cellular response to oxidative stress, endoplasmic reticulum (ER) stress, autophagy, and inflammation. Research has shown that oxidative stress can prompt an augmented Sesn2 expression in AP [31], aligning with the findings of our study. The NFE2 Like BZIP Transcription Factor 2 (Nfe2l2 or Nrf2), an intracellular transcriptional moderator, with Heme oxygenase 1 (Hmox1) being one of its key downstream effector molecules, plays a pivotal role in the body’s anti-inflammatory and antioxidative defense mechanisms. The efficacy of the Nrf2/Hmox1 signaling axis in mitigating AP has been substantiated by numerous studies [32–34]. Additionally, early peritoneal puncture drainage in a rat model with severe AP demonstrated a reduction in the disease’s severity, attenuating the oxidative stress through the activation of the Nrf2/Hmox1 pathway and the enhancement of autophagy [35]. The Mitogen activated protein kinase (MAPK) is integral to various oxidative stresses and inflammatory responses within the organism, asserting a significant regulatory influence over pancreatitis progression [36]. The crucial role of the MAPK pathway was further corroborated by our functional enrichment analysis. Recent studies have also uncovered that the Sesn2/MAPK/Nrf2/Hmox1 signaling cascade participates in autophagy regulation [37].

Previous studies have identified DEARGs under investigation as associated with AP. Mutations in KRAS proteins, which are prevalent in various cancer types, have been shown to mediate autophagy—a process consistently recognized for its role in cellular homeostasis. In Kras^*^G12D*^ mutant mice, a model of AP coupled with this gene mutation exhibited an exacerbated redox imbalance and heightened oxidative damage compared to those with the Kras wild-type genotype [38]. Overexpression of VMP1 has been shown to regulate autophagy during pancreatitis, thereby promoting pancreatic cell transformation via activation of the Kras gene [39]. Furthermore, the Cast/Calpastatin protein, a natural inhibitor of calpain (calcium-dependent cysteine protease), has been implicated in the development of AP through its dysregulation within the calpain system, as suggested by Heike Weber’s findings on caerulein-induced AP [40]. Additionally, the translocation of Cdkn1a, commonly known as p21, from the nucleus to the cytoplasm has been observed in experimental models of AP, playing a role in the phenotypic switch of myofibroblasts to fibroblasts [41]. There are also the NPC Intracellular Cholesterol Transporter 1 (NPC1) proteins, pivotal components within the limiting membrane of endosomes and lysosomes. Mutations in the Npc1 gene have been connected to lysosomal storage disorders, contributing to numerous degenerative diseases [42], yet little is known about their link to AP. Our recent results provide new insights to further explore the mechanisms of autophagy in AP.

To explore the potential association between key gene-drug interactions, we constructed a compound-protein interaction network and discovered that acetaminophen and vehicle emissions could interact with the mRNAs of all seven DEARGs. A subsequent comprehensive review of the existing literature revealed a correlation between the use of acetaminophen and the onset of acute pancreatitis. Several case reports have described that acetaminophen may trigger pharmacologically induced AP [43–45]. In the most recent revision of the Evidence-Based Classification of Pharmacological AP, Scott Tenner posits that the evidence implicating acetaminophen as a causative factor for AP is of moderate quality, based on case-control and pharmacoepidemiological studies [46]. Based on in vitro experiments, we also found that APAP induced a dose-dependent increase in amylase levels and affected the protein expression levels of seven biomarkers in acute pancreatitis. Understanding such associations is crucial for elucidating the pathogenic mechanisms underlying drug-induced pancreatitis, particularly in cases involving excessive use of acetaminophen.

Our study utilized four datasets from the GEO database, however, the limited sample size within each dataset potentially constrains the generalizability of our findings. Given the intricate regulation of autophagy, future research should elucidate the roles of pivotal genes across various stages and severities of AP. Moreover, the autophagy-related genes we identified displayed discrepancies between transcriptional and protein expression levels, indicating a need for additional investigations into their precise mechanisms. Furthermore, potential pharmacological agents that may influence the occurrence of AP were identified through bioinformatics analysis. However, it should be noted that molecular docking calculations primarily provide preliminary energy estimates and mode-of-action predictions, and subsequent in vitro binding assays (e.g., SPR or ITC) or functional validation are required for definitive affinity confirmation.

## Conclusion

Our research further elucidates the intimate link between autophagy and AP. We utilized four datasets from GEO database to identify and verification seven autophagy-related genes with differential expression associated with AP. Notably, Sesn2, Kras, Hmox1, and Nfe2l2 are highlighted as prospective therapeutic targets and biomarkers. Furthermore, it was discovered that these autophagy-related genes predominantly participate in the regulation of macroautophagy in AP. Additionally, our findings suggest that an excessive intake of acetaminophen may induce drug-related pancreatitis by facilitating autophagy.

## Supporting information

S1 Table Information on 212 autophagy-related genes in mice extracted from the Human Autophagy Database (HAMdb). (DOCX)

S2 Table Interaction actions and reference count of acetaminophen with mRNAs for the DEARGs. (DOCX)

S3 Table Interaction actions and reference count of vehicle emissions with mRNAs for the DEARGs. (DOCX)

S1 Fig Raw western blot images of differentially expressed autophagy-related genes (DEARGs) in tissues from animal models of acute pancreatitis. (PDF)

S2 Fig Raw western blot images of differentially expressed autophagy-related genes in AR42J cell of acute pancreatitis. (PDF)
