# Supplementary material for: Identification of autophagy-related key biomarkers in caerulein induced acute pancreatitis: In silico and in vivo study
Source: PLoS One. 2026 Mar 27;21(3):e0344110. doi: 10.1371/journal.pone.0344110 (PMC13028361; doi:10.1371/journal.pone.0344110)
Supplement: S1 Table — (DOCX) [file pone.0344110.s001.docx]

**S1 Table.** Information on 212 autophagy-related genes in mice extracted from the Human Autophagy Database (HAMdb).

| Symbol | HAMDB_ID | Molecule_Activity | | Effect_on | Disease_or_Function |
| --- | --- | --- | --- | --- | --- |
| ABHD5 | HAMDB842 | increased activity | increases | | autophagy of cells, autophagy of mitochondria |
| ACLY | HAMDB846 | decreased activity | increases | | autophagy |
| ACSL1 | HAMDB848 | decreased activity | decreases | | autophagy |
| ADIPOQ | HAMDB849 | decreased activity | increases | | autophagy of cardiomyocytes |
| AGT | HAMDB850 | increased activity | increases | | autophagy of cardiomyocytes |
| AGER | HAMDB851 | decreased activity | decreases | | autophagy of pancreatic cancer cell lines |
| AKT1 | HAMDB854 | decreased activity | increases | | autophagy of prostate cancer cell lines |
| AKT2 | HAMDB855 | decreased activity | increases | | autophagy of prostate cancer cell lines |
| AKT3 | HAMDB856 | decreased activity | increases | | autophagy of prostate cancer cell lines |
| AMBRA1 | HAMDB858 | increased activity | affects | | autophagy |
| APAF1 | HAMDB861 | decreased activity | increases | | autophagy of neurons |
| APOL1 | HAMDB862 | increased activity | increases | | autophagic cell death |
| APP | HAMDB864 | unknown change in activity | affects | | autophagy |
| ATG12 | HAMDB870 | increased activity | affects | | autophagy |
| ATG13 | HAMDB871 | increased activity | increases | | autophagy of fibroblast cell lines |
| ATG14 | HAMDB872 | increased activity | increases | | autophagy |
| ATG16L1 | HAMDB873 | decreased activity | affects | | autophagy |
| ATG3 | HAMDB876 | increased activity | affects | | macroautophagy of cells |
| ATG4A | HAMDB877 | increased activity | affects | | autophagy |
| ATG4B | HAMDB878 | increased activity | affects | | autophagy |
| ATG4C | HAMDB879 | increased activity | affects | | autophagy |
| ATG4D | HAMDB880 | increased activity | affects | | autophagy |
| ATG5 | HAMDB881 | increased activity | affects | | autophagy |
| ATG7 | HAMDB882 | increased activity | affects | | macroautophagy of cells |
| ATG9A | HAMDB882 | decreased activity | affects | | formation of autophagosomes |
| ATM | HAMDB882 | decreased activity | decreases | | accumulation of autophagic vacuoles |
| ATP13A2 | HAMDB882 | increased activity | affects | | autophagy |
| BAG6 | HAMDB882 | decreased activity | decreases | | autophagic cell death |
| BCL2 | HAMDB882 | decreased activity | decreases | | autophagy |
| BCL2L11 | HAMDB882 | decreased activity | decreases | | autophagy of macrophages |
| BECN1 | HAMDB882 | increased activity | increases | | autophagy of osteocytes |
| Becn2 | HAMDB882 | increased activity | affects | | autophagy of mitochondria |
| BMF | HAMDB884 | increased activity | affects | | autophagy |
| BNIP3 | HAMDB884 | decreased activity | affects | | autophagy |
| CAMK1 | HAMDB886 | increased activity | affects | | autophagy |
| CAMK4 | HAMDB886 | decreased activity | affects | | autophagy |
| CAPNS1 | HAMDB887 | decreased activity | affects | | autophagy |
| CASP1 | HAMDB891 | decreased activity | decreases | | autophagy |
| CASP2 | HAMDB891 | decreased activity | decreases | | autophagy of fibroblast cell lines |
| CASP8 | HAMDB896 | increased activity | affects | | autophagy |
| CASP9 | HAMDB896 | increased activity | decreases | | autophagy |
| CAST | HAMDB898 | decreased activity | increases | | autophagy of fibroblast cell lines |
| CDKN1A | HAMDB899 | increased activity | increases | | autophagy |
| CDKN1B | HAMDB900 | increased activity | affects | | autophagy |
| CDKN2A | HAMDB904 | decreased activity | increases | | autophagy of fibroblasts |
| CHMP4B | HAMDB904 | decreased activity | increases | | autophagy of epithelial cells |
| CHUK | HAMDB905 | increased activity | increases | | autophagy of fibroblast cell lines |
| CISD2 | HAMDB905 | increased activity | increases | | autophagy of muscle |
| CLEC11A | HAMDB905 | increased activity | increases | | autophagy of fibroblasts |
| CLEC12A | HAMDB913 | decreased activity | decreases | | autophagy of peritoneal macrophages |
| CLN3 | HAMDB913 | decreased activity | decreases | | autophagy of cells |
| CSF2 | HAMDB913 | decreased activity | decreases | | autophagy of macrophage cancer cell lines |
| CSHL1 | HAMDB913 | unknown change in activity | increases | | autophagy of macrophage cancer cell lines |
| CTSD | HAMDB914 | unknown change in activity | increases | | autophagy of peritoneal macrophages |
| DACT1 | HAMDB914 | decreased activity | decreases | | autophagy of peritoneal macrophages |
| DDIT4 | HAMDB914 | decreased activity | decreases | | autophagy of kidney cells |
| DENND3 | HAMDB918 | decreased activity | decreases | | autophagy of fibroblast cell lines |
| DNM1L | HAMDB919 | increased activity | affects | | autophagy |
| DUSP4 | HAMDB919 | decreased activity | increases | | autophagy of bone marrow-derived macrophages |
| EEF2 | HAMDB920 | decreased activity | increases | | autophagy of cortical neurons |
| EI24 | HAMDB921 | decreased activity | affects | | formation of autophagosomes |
| EIF2AK2 | HAMDB922 | decreased activity | decreases | | formation of autophagosomes |
| EIF2S1 | HAMDB923 | increased activity | affects | | formation of autophagosomes |
| ENG | HAMDB930 | decreased activity | increases | | autophagy of fibroblast cell lines |
| EP300 | HAMDB930 | decreased activity | increases | | autophagy of fibroblast cell lines |
| EPG5 | HAMDB931 | increased activity | increases | | autophagy of cardiomyocytes |
| EPM2A | HAMDB932 | increased activity | increases | | autophagy of cortical neurons, autophagy of mitochondria |
| EPO | HAMDB941 | increased activity | affects | | autophagy |
| ERBIN | HAMDB945 | decreased activity | decreases | | autophagy of fibroblasts |
| ERFE | HAMDB947 | increased activity | affects | | autophagy of mitochondria |
| FAS | HAMDB947 | decreased activity | increases | | autophagy |
| Fbxw7as1 | HAMDB948 | decreased activity | increases | | autophagy of cells |
| FLT3LG | HAMDB949 | decreased activity | affects | | autophagy |
| FOXO1 | HAMDB950 | increased activity | affects | | macroautophagy of cells |
| FOXO3 | HAMDB954 | decreased activity | increases | | autophagy of cells |
| GFER | HAMDB955 | increased activity | increases | | autophagy |
| GOPC | HAMDB964 | increased activity | increases | | autophagy of fibroblast cell lines |
| GSK3B | HAMDB972 | decreased activity | decreases | | autophagy of thymoma cell lines |
| HDAC1 | HAMDB972 | decreased activity | decreases | | autophagy of thymocytes |
| HDAC2 | HAMDB973 | increased activity | increases | | autophagy of fibroblast cell lines |
| HDAC6 | HAMDB977 | unknown change in activity | decreases | | autophagy of fibroblast cell lines, autophagy of mitochondria |
| HERC1 | HAMDB977 | decreased activity | increases | | autophagy of mitochondria |
| HIF1A | HAMDB980 | increased activity | decreases | | autophagy by muscle cell lines |
| HMGB1 | HAMDB980 | increased activity | decreases | | autophagy of heart |
| HMOX1 | HAMDB983 | decreased activity | decreases | | autophagy |
| HRAS | HAMDB986 | increased activity | affects | | autophagy |
| HTT | HAMDB987 | decreased activity | decreases | | autophagy of fibroblast cell lines |
| IDO1 | HAMDB987 | increased activity | increases | | autophagy of fibroblast cell lines |
| IFNG | HAMDB990 | increased activity | increases | | autophagy of fibroblast cell lines |
| IFNGR1 | HAMDB994 | decreased activity | decreases | | autophagy of embryonic cell lines, autophagy of endothelial cell lines |
| IGF1R | HAMDB995 | decreased activity | increases | | autophagy of muscle cells |
| IKBKB | HAMDB996 | increased activity | affects | | autophagy |
| IKBKG | HAMDB996 | decreased activity | decreases | | maturation of autophagosomes |
| IL11 | HAMDB998 | increased activity | increases | | macroautophagy of cells |
| IL17A | HAMDB999 | decreased activity | increases | | autophagy of cells |
| IL1B | HAMDB1000 | decreased activity | increases | | autophagy of keratinocytes |
| IL2 | HAMDB1001 | increased activity | decreases | | autophagy of hepatoma cell lines |
| IL3 | HAMDB1001 | increased activity | decreases | | autophagy of liver cells |
| IL33 | HAMDB1001 | increased activity | decreases | | formation of autophagosomes |
| IL6 | HAMDB1006 | decreased activity | affects | | macroautophagy of fibroblasts |
| INS | HAMDB1010 | increased activity | affects | | autophagy |
| Ins1 | HAMDB1014 | decreased activity | increases | | autophagy of cells |
| Irgm1 | HAMDB1016 | increased activity | increases | | autophagy of heart |
| KRAS | HAMDB1017 | increased activity | increases | | autophagy of fibroblasts |
| LAMP2 | HAMDB1017 | increased activity | increases | | autophagy of muscle |
| LEP | HAMDB1025 | decreased activity | increases | | autophagy of mitochondria |
| LMX1B | HAMDB1028 | increased activity | affects | | autophagy |
| LRRK2 | HAMDB1028 | decreased activity | decreases | | autophagy of fibroblast cell lines |
| MAF1 | HAMDB1032 | increased activity | affects | | autophagy |
| MAP1S | HAMDB1038 | decreased activity | decreases | | autophagy of fibroblast cell lines, autophagy of kidney cell lines |
| MAP2K4 | HAMDB1038 | decreased activity | decreases | | autophagy of fibroblasts |
| MAP2K7 | HAMDB1040 | increased activity | increases | | formation of autophagosomes |
| Map3k7 | HAMDB1042 | increased activity | increases | | formation of autophagosomes |
| MAPK14 | HAMDB1043 | increased activity | increases | | fusion of autophagosomes |
| MAPK8 | HAMDB1044 | increased activity | decreases | | autophagy |
| MBOAT4 | HAMDB1046 | increased activity | increases | | autophagy |
| MCL1 | HAMDB1046 | increased activity | increases | | macroautophagy of cells |
| MFN2 | HAMDB1048 | unknown change in activity | increases | | autophagy of fibroblast cell lines |
| MFSD8 | HAMDB1049 | increased activity | decreases | | macroautophagy of cells |
| Mt3 | HAMDB1049 | increased activity | increases | | macroautophagy of cells |
| MTOR | HAMDB1050 | unknown change in activity | increases | | autophagy of fibroblasts |
| MYC | HAMDB1058 | unknown change in activity | affects | | autophagy |
| MYD88 | HAMDB1060 | decreased activity | affects | | autophagy |
| MYOCD | HAMDB1061 | increased activity | increases | | autophagy of bone marrow-derived macrophages |
| NFE2L2 | HAMDB1061 | increased activity | increases | | autophagy of macrophage cancer cell lines |
| NHLRC1 | HAMDB1061 | increased activity | increases | | autophagy of gastric epithelial cells |
| NLRC4 | HAMDB1061 | increased activity | increases | | autophagy of fibroblast cell lines |
| NLRP6 | HAMDB1062 | decreased activity | decreases | | autophagy of cells |
| NLRX1 | HAMDB1064 | decreased activity | decreases | | accumulation of autophagic vacuoles |
| NPC1 | HAMDB1067 | decreased activity | decreases | | autophagy of hepatocytes |
| NR1D1 | HAMDB1067 | unknown change in activity | increases | | autophagy of fibroblast cell lines |
| NRBF2 | HAMDB1067 | decreased activity | decreases | | autophagy of fibroblasts |
| NUPR1 | HAMDB1068 | unknown change in activity | increases | | autophagy of fibroblast cell lines |
| OPTN | HAMDB1068 | increased activity | increases | | macroautophagy of cells |
| OSTM1 | HAMDB1068 | decreased activity | decreases | | autophagy of fibroblasts |
| PARK2 | HAMDB1069 | decreased activity | increases | | autophagy of cells |
| PIK3C2A | HAMDB1070 | decreased activity | increases | | autophagy |
| PIK3C2B | HAMDB1070 | increased activity | decreases | | autophagy of lung cell lines |
| PIK3C3 | HAMDB1070 | decreased activity | increases | | autophagic cell death |
| PIK3R1 | HAMDB1071 | increased activity | affects | | autophagy of gastric epithelial cells |
| PIM2 | HAMDB1071 | increased activity | increases | | autophagy of macrophages |
| PINK1 | HAMDB1072 | increased activity | increases | | autophagy of liver tissue |
| PLA2G4A | HAMDB1074 | decreased activity | increases | | autophagy of cells |
| PLD1 | HAMDB1075 | decreased activity | decreases | | autophagy |
| POLDIP2 | HAMDB1076 | increased activity | increases | | autophagy of pancreatic cancer cell lines |
| POLG | HAMDB1077 | increased activity | decreases | | autophagy of muscle |
| PPARG | HAMDB1078 | increased activity | decreases | | autophagy of Cytoplasm |
| PRKAA1 | HAMDB1080 | decreased activity | increases | | autophagy of cells |
| PRKAA2 | HAMDB1089 | unknown change in activity | affects | | autophagy of keratinocytes |
| PRKCD | HAMDB1089 | unknown change in activity | increases | | autophagy |
| PRNP | HAMDB1093 | increased activity | affects | | autophagy |
| PSEN1 | HAMDB1093 | increased activity | affects | | chaperone mediated autophagy |
| PYCARD | HAMDB1097 | increased activity | decreases | | autophagy |
| RAB12 | HAMDB1100 | increased activity | affects | | autophagy |
| RALGAPB | HAMDB1102 | increased activity | increases | | autophagy |
| RB1 | HAMDB1102 | decreased activity | affects | | autophagy |
| RB1CC1 | HAMDB1104 | decreased activity | increases | | autophagy |
| RHEB | HAMDB1107 | decreased activity | affects | | autophagy |
| RIPK1 | HAMDB1107 | decreased activity | increases | | accumulation of autophagosomes |
| RNASEL | HAMDB1110 | decreased activity | decreases | | autophagic cell death of fibroblast cell lines |
| RNF152 | HAMDB1111 | decreased activity | decreases | | autophagic cell death of fibroblast cell lines |
| RNF5 | HAMDB1111 | decreased activity | increases | | autophagic cell death of fibroblast cell lines |
| RNF7 | HAMDB1112 | increased activity | increases | | macroautophagy of cells |
| ROCK1 | HAMDB1112 | decreased activity | decreases | | autophagy of fibroblasts |
| ROCK2 | HAMDB1113 | decreased activity | increases | | autophagy |
| RRAGA | HAMDB1114 | increased activity | increases | | autophagic cell death of fibroblast cell lines |
| RYR2 | HAMDB1114 | decreased activity | decreases | | autophagic cell death of fibroblast cell lines |
| S1PR3 | HAMDB1116 | decreased activity | decreases | | autophagy |
| SCD | HAMDB1117 | decreased activity | decreases | | autophagy of heart |
| SERPINA1 | HAMDB1117 | decreased activity | increases | | autophagy of fibroblast cell lines |
| SESN2 | HAMDB1125 | decreased activity | decreases | | autophagy of fibroblast cell lines |
| SH3GLB1 | HAMDB1125 | decreased activity | decreases | | autophagy of fibroblast cell lines |
| SIRT1 | HAMDB1126 | increased activity | affects | | autophagy |
| SLC33A1 | HAMDB1130 | increased activity | decreases | | autophagy |
| SMAD2 | HAMDB1133 | increased activity | affects | | autophagy |
| SNCA | HAMDB1133 | increased activity | decreases | | autophagy |
| SOAT1 | HAMDB1133 | increased activity | decreases | | macroautophagy of cells |
| SOGA1 | HAMDB1133 | increased activity | increases | | autophagy of Cytoplasm |
| SPTLC1 | HAMDB1133 | decreased activity | increases | | autophagy of cells |
| SPTLC2 | HAMDB1133 | increased activity | increases | | autophagy of peritoneal macrophages |
| SPTSSA | HAMDB1133 | decreased activity | increases | | autophagy |
| SREBF2 | HAMDB1135 | increased activity | affects | | autophagy |
| SRPX | HAMDB1136 | decreased activity | decreases | | autophagy of Cytoplasm, autophagy of macrophage cancer cell lines |
| STAT1 | HAMDB1138 | decreased activity | increases | | autophagy of smooth muscle cells |
| TAS1R3 | HAMDB1147 | decreased activity | increases | | autophagy of keratinocytes |
| TBK1 | HAMDB1147 | increased activity | increases | | macroautophagy |
| TCIRG1 | HAMDB1149 | decreased activity | affects | | autophagy |
| TECPR1 | HAMDB1150 | decreased activity | increases | | autophagy of bone marrow-derived macrophages |
| THPO | HAMDB1152 | increased activity | affects | | autophagy |
| TICAM1 | HAMDB1153 | decreased activity | decreases | | autophagy of peritoneal macrophages |
| TICAM2 | HAMDB1153 | decreased activity | decreases | | autophagy of fibroblast cell lines |
| TLR1 | HAMDB1158 | increased activity | affects | | autophagy |
| TLR3 | HAMDB1158 | increased activity | decreases | | macroautophagy of cells |
| TLR4 | HAMDB1160 | decreased activity | increases | | autophagy of muscle |
| TLR5 | HAMDB1161 | increased activity | affects | | autophagy |
| TLR6 | HAMDB1163 | decreased activity | increases | | autophagy |
| TLR7 | HAMDB1163 | decreased activity | decreases | | autophagy of fibroblast cell lines |
| TMBIM6 | HAMDB1165 | unknown change in activity | increases | | autophagy by retinal ganglion cells |
| TOP1MT | HAMDB1166 | decreased activity | increases | | accumulation of autophagosomes |
| TP53 | HAMDB1168 | decreased activity | decreases | | autophagy of mitochondria |
| TP53INP2 | HAMDB1168 | decreased activity | decreases | | autophagy |
| TPCN2 | HAMDB1168 | increased activity | increases | | autophagy of cortical neurons, autophagy of mitochondria |
| TSC2 | HAMDB1168 | decreased activity | decreases | | autophagy of heart, autophagy of mitochondria |
| UBR4 | HAMDB1168 | decreased activity | affects | | autophagy |
| ULK1 | HAMDB1174 | increased activity | affects | | macroautophagy of cells |
| ULK2 | HAMDB1175 | increased activity | affects | | macroautophagy of cells |
| VMP1 | HAMDB1176 | increased activity | affects | | macroautophagy of cells |
| VPS18 | HAMDB1176 | decreased activity | decreases | | autophagy of CD4+ T-lymphocytes |
| VTI1B | HAMDB1176 | increased activity | affects | | autophagy |
| WASHC1 | HAMDB1176 | decreased activity | decreases | | autophagy of T lymphocytes |
| XBP1 | HAMDB1176 | decreased activity | decreases | | autophagy of cells |
| ZC3H12A | HAMDB1179 | increased activity | increases | | autophagy of macrophage cancer cell lines |
