# Supplementary material for: Identification of autophagy-related key biomarkers in caerulein induced acute pancreatitis: In silico and in vivo study
Source: PLoS One. 2026 Mar 27;21(3):e0344110. doi: 10.1371/journal.pone.0344110 (PMC13028361; doi:10.1371/journal.pone.0344110)
Supplement: S2 Table — (DOCX) [file pone.0344110.s002.docx]

**Table S2.** Interaction actions and reference count of acetaminophen with mRNAs for the DEARGs.

| Chemical Name | Chemical ID | Gene Symbol | Interaction Actions | Reference Count |
| --- | --- | --- | --- | --- |
| Acetaminophen | D000082 | **CDKN1A** | Affects expression | **2** |
| Acetaminophen | D000082 | **CDKN1A** | Increases expression | **7** |
| Acetaminophen | D000082 | **CDKN1A** | Increases expression | **2** |
| Acetaminophen | D000082 | **CAST** | Affects expression | **1** |
| Acetaminophen | D000082 | **SESN2** | Decreases expression | **1** |
| Acetaminophen | D000082 | **SESN2** | Affects expression | **1** |
| Acetaminophen | D000082 | **NPC1** | Affects expression | **1** |
| Acetaminophen | D000082 | **KRAS** | Affects expression | **1** |
| Acetaminophen | D000082 | **NFE2L2** | Affects expression | **1** |
| Acetaminophen | D000082 | **NFE2L2** | Decreases expression | **1** |
| Acetaminophen | D000082 | **NFE2L2** | Affects localization | **2** |
| Acetaminophen | D000082 | **NFE2L2** | Decreases expression | **1** |
| Acetaminophen | D000082 | **NFE2L2** | Increases activity  Increases localization | **1** |
| Acetaminophen | D000082 | **NFE2L2** | Increases activity | **3** |
| Acetaminophen | D000082 | **NFE2L2** | Increases expression | **2** |
| Acetaminophen | D000082 | **NFE2L2** | Increases localization | **1** |
| Acetaminophen | D000082 | **NFE2L2** | Increases activity  Increases expression  Increases localization | **1** |
| Acetaminophen | D000082 | **NFE2L2** | Increases activity  Increases expression  Increases localization | **1** |
| Acetaminophen | D000082 | **NFE2L2** | Increases activity  Increases expression  Increases localization | **1** |
| Acetaminophen | D000082 | **NFE2L2** | Increases expression | **3** |
| Acetaminophen | D000082 | **HMOX1** | Affects expression | **3** |
| Acetaminophen | D000082 | **HMOX1** | Decreases expression | **2** |
| Acetaminophen | D000082 | **HMOX1** | Increases expression | **17** |
| Acetaminophen | D000082 | **HMOX1** | Increases expression | **5** |
