# Supplementary material for: Identification of autophagy-related key biomarkers in caerulein induced acute pancreatitis: In silico and in vivo study
Source: PLoS One. 2026 Mar 27;21(3):e0344110. doi: 10.1371/journal.pone.0344110 (PMC13028361; doi:10.1371/journal.pone.0344110)
Supplement: S3 Table — (DOCX) [file pone.0344110.s003.docx]

**Table S3.** Interaction actions and reference count of vehicle emissions with mRNAs for the DEARGs.

| **Chemical Name** | Chemical ID | **Gene Symbol** | Interaction Actions | **Reference Count** |
| --- | --- | --- | --- | --- |
| **Vehicle Emissions** | D001335 | **NPC1** | Decreases methylation | **1** |
| **Vehicle Emissions** | D001335 | **SESN2** | Decreases methylation | **1** |
| **Vehicle Emissions** | D001335 | **CAST** | Affects methylation | **1** |
| **Vehicle Emissions** | D001335 | **HMOX1** | Decreases reaction  Increases expression | **1** |
| **Vehicle Emissions** | D001335 | **HMOX1** | Affects response to substance  Increases expression  Increases reaction | **1** |
| **Vehicle Emissions** | D001335 | **HMOX1** | Decreases response to substance | **1** |
| **Vehicle Emissions** | D001335 | **HMOX1** | Decreases reaction  Increases expression | **1** |
| **Vehicle Emissions** | D001335 | **HMOX1** | Increases expression | **2** |
| **Vehicle Emissions** | D001335 | **HMOX1** | Increases expression | **2** |
| **Vehicle Emissions** | D001335 | **HMOX1** | Increases expression  Increases reaction | **1** |
| **Vehicle Emissions** | D001335 | **HMOX1** | Decreases expression | **1** |
| **Vehicle Emissions** | D001335 | **HMOX1** | Decreases methylation | **1** |
| **Vehicle Emissions** | D001335 | **HMOX1** | Affects expression  Increases abundance | **1** |
| **Vehicle Emissions** | D001335 | **HMOX1** | Increases abundance  Increases expression | **2** |
| **Vehicle Emissions** | D001335 | **HMOX1** | Increases expression | **8** |
| **Vehicle Emissions** | D001335 | **HMOX1** | Increases expression | **3** |
| **Vehicle Emissions** | D001335 | **CDKN1A** | Affects methylation | **1** |
| **Vehicle Emissions** | D001335 | **NFE2L2** | Affects localization  Decreases reaction | **1** |
| **Vehicle Emissions** | D001335 | **NFE2L2** | affects response to substance  increases expression  increases reaction | **1** |
| **Vehicle Emissions** | D001335 | **NFE2L2** | Decreases reaction  Increases expression | **1** |
| **Vehicle Emissions** | D001335 | **NFE2L2** | Decreases reaction  Increases expression | **1** |
| **Vehicle Emissions** | D001335 | **NFE2L2** | Affects expression | **1** |
| **Vehicle Emissions** | D001335 | **NFE2L2** | Affects localization  Increases expression | **1** |
| **Vehicle Emissions** | D001335 | **NFE2L2** | Increases expression | **1** |
| **Vehicle Emissions** | D001335 | **NFE2L2** | Affects expression  Increases abundance | **1** |
| **Vehicle Emissions** | D001335 | **NFE2L2** | Increases abundance  Increases expression | **1** |
| **Vehicle Emissions** | D001335 | **NFE2L2** | Increases abundance  Increases expression | **1** |
| **Vehicle Emissions** | D001335 | **NFE2L2** | Increases expression | **2** |
| **Vehicle Emissions** | D001335 | **NFE2L2** | Increases expression | **1** |
| **Vehicle Emissions** | D001335 | **NFE2L2** | Increases methylation | **1** |
| **Vehicle Emissions** | D001335 | **KRAS** | Increases methylation | **1** |
