# Supplementary figures and images for: Identification of autophagy-related key biomarkers in caerulein induced acute pancreatitis: In silico and in vivo study
Source: PLoS One. 2026 Mar 27;21(3):e0344110. doi: 10.1371/journal.pone.0344110 (PMC13028361; doi:10.1371/journal.pone.0344110)

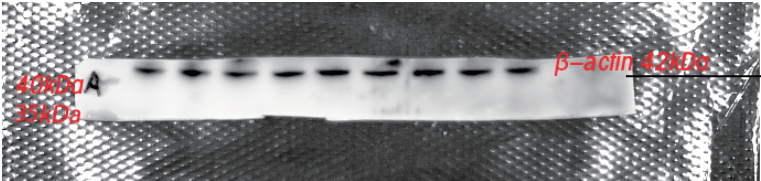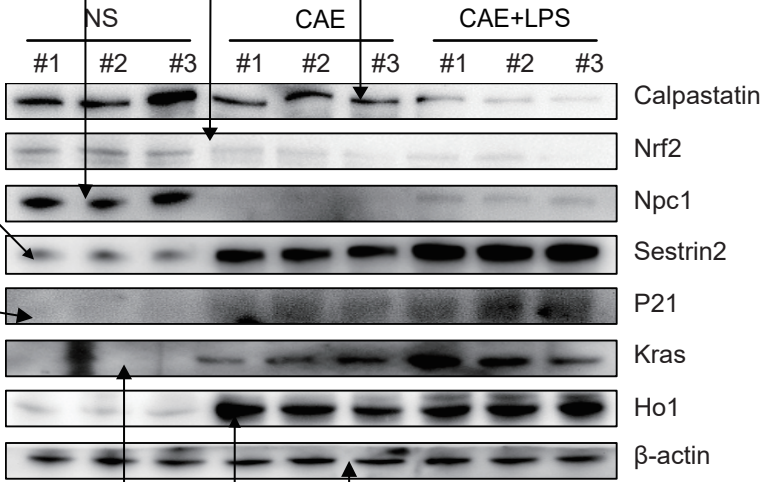

Supplement: S1 Fig — (PDF) [file pone.0344110.s004.pdf]

AR42J

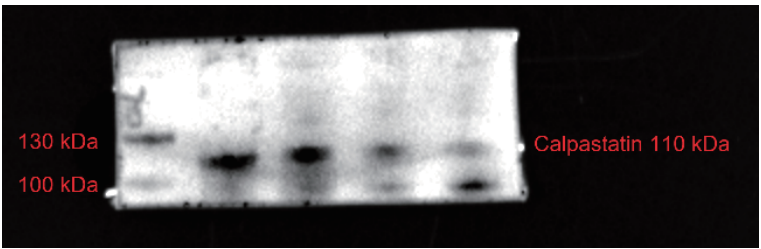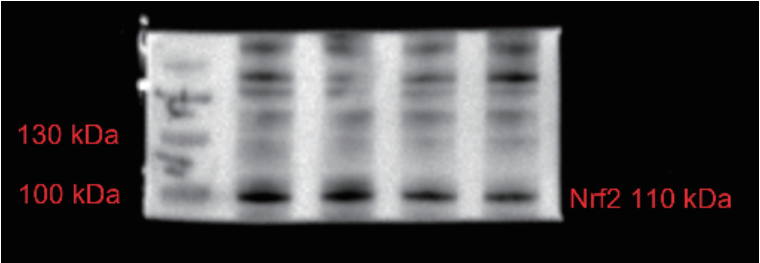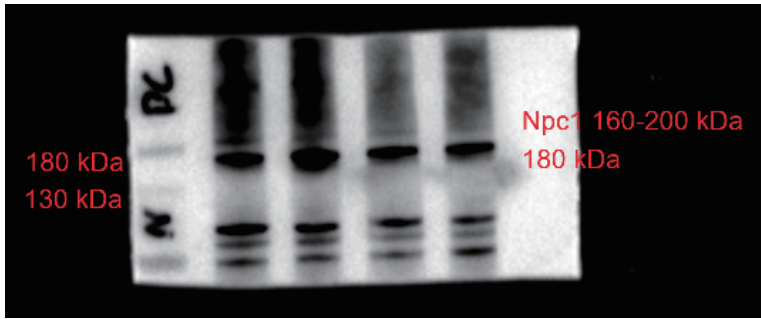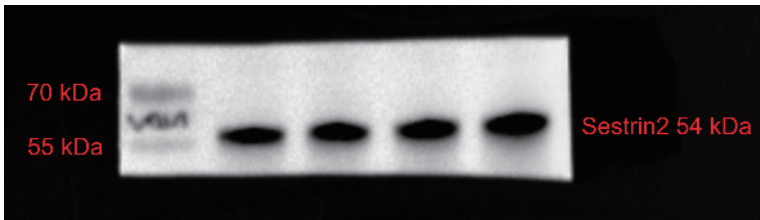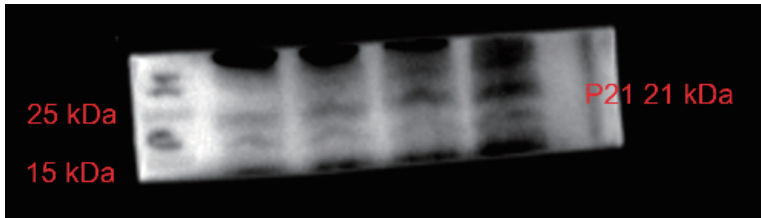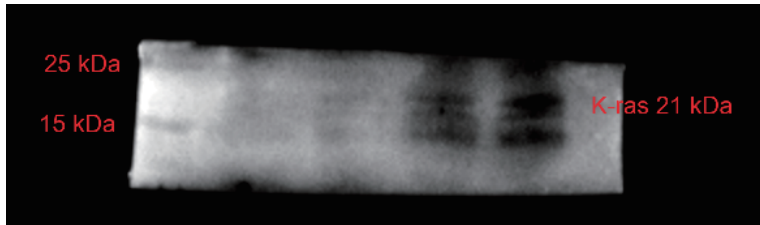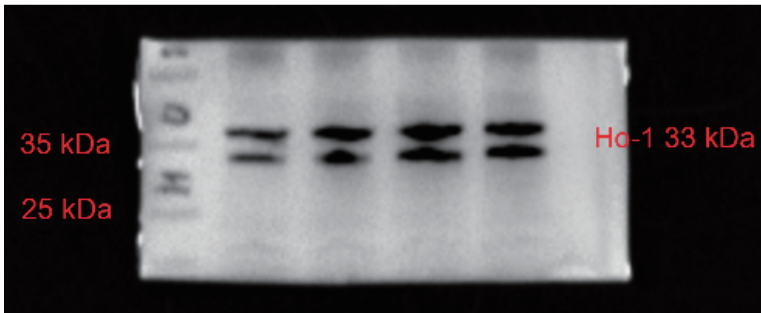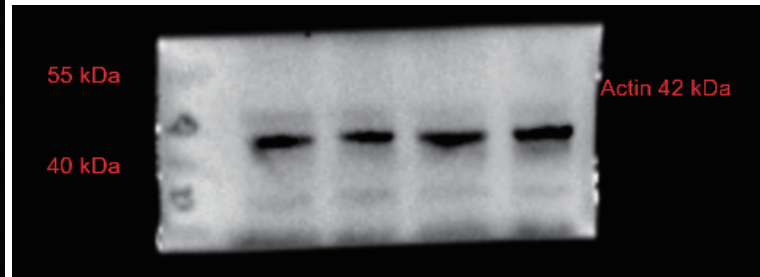

Supplement: S2 Fig — (PDF) [file pone.0344110.s005.pdf]
